# Effect of Different Suprahepatic Vena Cava Reconstruction Methods on the Hemodynamics of Rats after Liver Transplantation

**DOI:** 10.1371/journal.pone.0072695

**Published:** 2013-09-04

**Authors:** Hongdong Wang, Chonghui Li, Jianjun Hu, Hongbin Xu, Xu Ji, Xiaofeng Wang, Xuedong Wang, Yukun Luo, Hailin Li, Kesen Xu, Sheng Ye, Aiqun Zhang, Jiahong Dong

**Affiliations:** 1 Department of Hepatobiliary Surgery, Qilu Hospital of Shandong University, Jinan, Shandong Province, China; 2 Hospital and Institute of Hepatobiliary Surgery, PLA General Hospital, Beijing, China; 3 Department of Hepatobiliary Surgery, 302 Hospital of PLA, Beijing, China; 4 Department of Ultrasound, PLA General Hospital, Beijing, China; The University of Hong Kong, Hong Kong

## Abstract

**Background:**

There are few studies on the hemodynamic changes after orthotopic liver transplantation in rats. In this study, we aimed to evaluate the effect of different suprahepatic vena cava (SHVC) reconstruction methods on the hemodynamics of rats after liver transplantation.

**Materials and Methods:**

Three rat liver transplantation groups were created according to the SHVC reconstruction method: Kamada’s two-cuff technique, a modified veno-lined stent technique, and Harihara’s three-cuff technique. Ten rats of similar weight were grouped as the control. Anatomical, ultrasonic, and hemodynamic parameters and the microcirculation of the liver were measured after transplantation. The detailed operation time, operative complications, and animal survival were recorded.

**Results:**

All the recipients showed portal hypertension one month after transplantation. The portal hypertension in the group with the modified veno-lined stent technique was the most severe. The value measured with real-time elastography was significantly higher in the recipients using the modified veno-lined stent technique than in the other two groups (P<0.01). There was no difference in the graft microcirculation after reperfusion among the three groups. The survival rate of the three groups displayed no difference, but the modified veno-lined stent technique led to more venous complications than the other two techniques.

**Conclusions:**

The hemodynamics after liver transplantation in rats is determined not only by the cuff used for portal vein reconstruction but also by the cuff or stent for the SHVC. Some SHVC reconstruction methods, such as the modified veno-lined stent technique, Miyata’s or Settaf’s three-cuff techniques, significantly affect the hemodynamics.

## Introduction

Over 30 years have passed since Miyata [1] published his three-cuff method for rat orthotopic liver transplantation (ROLT). Numerous modifications have continued to emerge. Initially, Miyata and Settaf [2] used the thoracic segment of the vena cava to cover the cuff. Subsequently, Tsuchimfoto [3] and Harihara [4] introduced the abdominal portion of the vena cava. In 2005, Tan et al. [5] invented a veno-lined stent for the reconstruction of the suprahepatic vena cava (SHVC), which shortens the length of the anhepatic time. They reported that their method had obtained a similar survival rate as the classical two-cuff method developed by Kamada and Calne [6].

Although these improvements can optimize the operation, the challenges of cuff inserting and ligation remain. For example, usage of the donor’s thoracic SHVC (TSHVC) ensures difficulty in covering the cuff because of TSHVC’s poor distensibility, especially the lower part proximate to the diaphragm. Alternatively, the abdominal portion of the SHVC can be selected to cover the cuff. This procedure remains difficult because the venous walls are very short, although the anterior wall is relatively longer than the posterior. The diaphragm crosses the abdominal SHVC (ASHVC) in an inclined plane, not horizontally. This situation ensures that the ligation of the recipient’s SHVC is very difficult. The problems of the cuff technique presented above can be resolved with Tan’s method, using a veno-lined stent.

To some extent, the three-cuff technique simplifies the ROLT procedure, while the two-cuff method facilitates reliable reconstruction of the SHVC. Both techniques led to a hemodynamic disturbance in rats. Kuznetsova and Imamura’s studies showed the following hemodynamic changes: decrease in the portal vein (PV) blood flow, a slight increase in the portal pressure and marked Porto systemic shunts [7], [8]. These hemodynamic changes present regardless of the arterialization of the graft liver [9]. Kuznetsova finds that running suture of all the veins will not show portal hypertension [7]. Imamura considers that the portal hypertension of the two-cuff method is most likely caused by the portal cuff [8].

There has been no study of whether the suprahepatic cuff will disturb the hemodynamics, and no further application or evaluation of the veno-lined stent technique for ROLT was reported. We investigated the hemodynamic parameters of the rats in which SHVC were reconstructed by the veno-lined stent technique, Kamada’s microsuture technique and Harihara’s cuff technique.

## Materials and Methods

### Animals

Male Sprague-Dawley rats (320–350 g) were obtained from the Animal Center, Academy of Military Medical Sciences of the PLA, Beijing, China. They were housed under specific pathogen-free conditions with a 12-hour light/dark cycle and were fasted 24 hours before the operation.

This experiment was carried out strictly in accordance with the ARRIVE guidelines for animal studies [10]. The experimental procedures were approved by the Institutional Animal Care and Use Committee of the Chinese PLA General Hospital. The animals were treated humanely and received good care. Every effort was made to minimize the suffering of the animals. The surgical or invasive procedures were performed under deep anesthesia by ether inhalation. To prevent occasional apnea caused by respiratory secretions, atropine sulfate (8 µg/Kg i.m.) was administered to each animal. After the operations, the animals were monitored every 12 hrs. Fatal complications during one month of observation resulted in the animal being sacrificed without suffering by administration of chloral hydrate. These manifestations include marked loss of weight, serious loss of hair, jaundice or shock. At the end of the observation period, all the animals were sacrificed using chloral hydrate to obtain the specimens (see “Histological Assessment” below).

### Experimental Design

According to the different methods used for the reconstruction of SHVC, three liver transplantation groups were created. In group 1, the method described by Kamada method was used. In group 2, the veno-lined stent technique developed by Tan was used. As a modification, the TSHVC of the donor was used to cover the lower end of the stent. In group 3, the SHVC was reconstructed with Harihara’s cuff technique [4]. The ASHVC of the donor was cuffed and inserted into the ASHVC of the recipient. The transplantations were performed by the same surgeon who had expertise in performing ROLT. To exclude any bias from the effect of training, all three types of transplantations were performed each day, and the sequences of the three types of transplantations were randomly arranged.

The time expended on each step were exactly measured in each pair of transplantations. The animals that survived 24 hours were defined as successful operations. Twelve recipients in each group were used for survival analysis. Other recipients that survived 24 hours, 72 hours and one month in each group were used for the hemodynamic study. Six survivors were used to measure the pressure and blood flow of PV and IHVC in each observation time point in each group.

In addition, 10 normal rats were used as control. Sham operations were performed on these animals. An anatomical study of the transplantation related vessels was performed. Additional procedures, such as real-time elastosonography, laser speckle perfusion imaging, hemodynamic study and histological assessment, were performed on the control group and the surviving recipients.

### Liver Transplantation

The cuffs for the PV, IHVC, and ASHVC were 6 F (2.2 mm× 2.8 mm), 8 F (3.0 mm × 3.6 mm) and 11 F (4 mm × 5 mm) polyethylene tubes. An operating microscope (Binocular Operation Microscope; Type GX.SS.22-3; Shanghai Medical Optical Instruments Co., Ltd, China) was used during the operation. Most of the procedures were based on Kamada’s two-cuff technique [6] except for the reconstruction method of SHVC. The following descriptions present the details of our modifications of Kamada’s method and the veno-lined stent technique.

### Donor Operation

For all the donors, the splenic vein was never ligated as the section of the PV superior to this vessel was long enough to be anastomosed. All the cuffs were fixed onto the IHVC, the PV or the SHVC during the graft perfusion.

**Figure 1:**
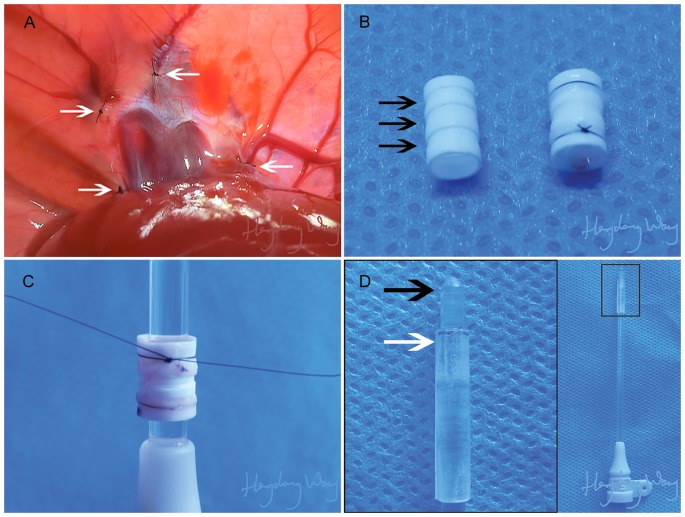
Details of the modified veno-lined stent technique. **A.** All of the four phrenic veins (white arrow) of the graft liver were pierced and ligated reliably. **B.** The modified three-groove stent used in the modified veno-lined stent technique (left, black arrow for groove). The donor’s IHVC segment between the left renal vein and the left iliolumbar vein was covered over the stent and ligated on both ends (right). **C.** A polymethylmethacrylate stick was used to hold the vessel in place and to prevent torsion of the vessel. **D.** The modified propeller with a blunt head (black arrow) and an annular base (white arrow).

In the modified veno-lined stent technique group (group 2), four phrenic veins were pierced and ligated. The phrenic ring and a 4 mm long TSHVC was reserved (Figure 1A).

The graft harvest was completed with the insertion and fixation of a 24 G polyethylene tube into the common hepatic artery. The liver was stored in an ice cold saline bath until transplantation.

### Preparation of Veno-lined Stent and Propeller

After completion of the graft harvesting, the IHVC between the left renal vein and the left iliolumbar vein was flushed and freed according to Tan’s method [5].

A stent was constructed with 6 F polyethylene tube (inside diameter 2.2 mm, outside diameter 2.8 mm; Shenzhen Ant Hi-Tech Industrial Co., Ltd.). For a modification, three parallel and transverse grooves were carved on the surface of the stent at uniform intervals (Figure 1B).

When the three-groove stent was slipped over the IHVC, the upper end of the IHVC was everted over the top groove of the stent and fixed with a circumferential 8-0 monofilament thread. The IHVC was cut off at 1-cm-lengths and passed through by a polymethylmethacrylate stick (diameter 1.8 mm), which was fixed in a piece of plasticine (Figure 1C). The lower end of the IHVC was ligated over the bottom groove as was the upper end of the IHVC. The stent was stored in the identical ice-cold saline as the graft liver. A modified stent propeller was made by the same polymethylmethacrylate stick, which had a blunt head and an annular base (Figure 1D).

### Recipient Operation

In the recipient, the majority of the procedures for mobilizing the original liver were similar to Kamada’s method [6], except for the following techniques. First, the PV and IHVC were mobilized only in the trunk. The anterior wall of the PV was cut in the bifurcation and then the posterior wall. The length of the PV could be gained as long as possible. Second, a small piece of the right lower lobe was reserved when the IHVC was cut off, and the ligated right adrenal vein was also reserved. This procedure could make the anastomosis easier because the IHVC was gained as long as possible and the posterior wall of the IHVC was well exposed by the distraction of the reserved adrenal vein.

In addition to the small tips for the ROLT, which were mentioned above, additional procedures were recommended as modifications of Tan’s veno-lined stent technique. The middle and left lateral lobes were resected using Higgins’ method [11], and the dorsal ligaments of the inferior vena cava were completely dissected before the IHVC and the PV were clamped. When the donor’s TSHVC was covered over the stent’s lower groove, two microforceps were used to clamp the top and middle groove in a cross position. This action would retain the stent in a stable position and make the anastomosis simple.

### Real-Time Elastography

The real-time elastography was a relatively new method for the measurement of tissue elasticity. It has been used in clinical medicine in recent years for evaluating hepatic fibrosis and portal hypertension by measuring liver stiffness [12], [13], [14], [15], [16], [17].

In our experiment, real-time elastography was performed for the control and the recipients that survived one month after liver transplantation. The hepatic elasticity was measured using a shear-wave elastosonography (AixPlorer model, Supersonic Imagine, Aix en Provence, France) coupled with a convex array probe (3–6 MHz). The regions of interest were placed on the median lobe, which was located by the median hepatic vein. At least three regions of interest were measured in each animal. The median elastic ratios of these regions of interest were immediately calculated using Supersonic Imagine.

### Laser Speckle Perfusion Imaging

Laser speckle perfusion imaging (LSPI) could noninvasively map the surface blood flow of tissues [18]. For the control group, the animals underwent ether anesthesia in the prone position after real–time elastography was completed. Laparotomy was performed through a midline incision. LSPI was performed with the FLPI-2 (Full-Field Laser Perfusion Imager; Moor Instruments, Essex, UK) in the low resolution/high speed setting at a display rate of 25 Hz, time constant of 1.0 s, and camera exposure time of 20 ms. For the recipients, the laser speckle perfusion images were performed 3, 10 and 20 min after the reperfusion of the graft liver. The LSPI was performed in an operating room with a constant temperature of 28°C. And the scanning distance was set at 30 cm.

### Anatomic Study of the Transplantation-Related Vessels

To define the precise size of the cuffs and stents used in this experiment, the control group was used for studying the anatomies of the transplantation**-**related vessels with Blain’s method [19] immediately following completion of the laser speckle perfusion images.The vessels were carefully dissected and precisely measured in situ. The outer diameter, the length and the branches of each vessel were recorded. The hemodynamic studies were performed on the animals in the control group for measuring the normal value.

### Measurement of the Hemodynamics of the IHVC and PV

The IHVC between the two renal veins and the PV between the gastroduodenal vein and the splenic vein were carefully dissected. One percent lidocaine was used topically to release vasospasm. The IHVC and PV blood flow were measured by a Transonic Flow Probe (TS420 Transit-time Perivascular Flowmeter, Transonic Systems Inc., Ithaca, New York, USA). The pressure of the IHVC and PV were continuous monitored for 5 min by a Multichannel Physiological Record Instrument (Type RM6240B, Chendu Instrument Factory, China). In addition to the normal value in the control group, we measured the hemodynamic parameters at 24 hrs, 72 hrs and 30 days in the transplantation groups.

### Histological Assessment

One month after the orthotopic liver transplantation, the animals in the control group and the survivors of the three transplantation groups were sacrificed by a chloral hydrate overdose. The liver and spleen were removed and weighed. The liver was fixed in formalin for the histological analysis. The liver specimens were obtained from the median lobe. After being embedded in paraffin, the specimens were cut into 4-µm-thick sections and stained with hematoxylin and eosin. To perform the semi-quantitative analysis, the scoring system introduced by Zhao et al. [20] and Imamura [8] was applied with some modifications. There were four items for evaluating the histological parameters: cellular infiltration and sinusoidal congestion, which was graded from 0 to 3; ductular proliferation and septal fibrosis, which were graded from 0 to 5. The grading methods for sinusoidal congestion were modified as follows: 0: none; 1: minimal (erythrocytes congested in the minor hepatic sinusoid); 2: moderate (most of the hepatic sinusoid showing involvement but without atrophy or necrosis of the hepatocytes); 3: severe (almost all of the sinusoid showing involvement with the atrophy or necrosis of the hepatocytes). All the histological analyses were performed by the same researcher using a blinding method.

### Statistical Analysis

The quantitative data are presented as the mean ± SDs, and the groups were compared using the t-test, Chi-squared test and one-way ANOVA. The survival data were compared by log-rank analysis using the Kaplan and Meier method. P<0.05 was considered to have statistical significance. All of the analyses were performed using SPSS software, version 18 (SPSS, Inc., Chicago, IL, USA).

## Results

### Anatomical Measurements of Veins in the Normal Rats

In the control group, the anatomical parameters of the transplantation-related veins were measured. These veins included the abdominal and thoracic segments of the SHVC, the PV and IHVC, which were used for connecting the cuffs and the IHVC, which was used for covering the stent.

Among these veins, the IHVC for covering the stent had 2–3 branches and the ASHVC had four prominent phrenic veins, which distribute in the left corner of the lower end of the SHVC and in the right corner and right anterior wall of the upper end of the SHVC.

The IHVC for covering the stent was slightly wider than the TSHVC. The 6 F polyethylene tube was appropriate for making the stent. Regarding the PV and IHVC, the 5 F and 8 F tubes were wide enough, while a wider polyethylene tube (inner diameter 4 mm, outer diameter 5 mm) was used for the reconstruction of the ASHVC in Harihara’s method (Table 1).

**Table 1:** Anatomical Study of the Liver Transplantation-Related Vessels.

Vessels	Middle Diameter (mm) (Mean ± SD)	Length (mm) (Mean ± SD)	No. of Branches
IHVC*	4.48±0.20	6.20±0.56	1
IHVC#	3.13±0.18	12.41±0.82	2–3
PV	2.43±0.23	3.54±0.18	0
TSHVC	2.82±0.12	12.87±0.79	0
ASHVC	6.76±0.67	3.57±0.11	4

* the IHVC segment between the right lower lobe and the right renal vein;

# the IHVC segment between the left renal vein and the left iliolumbar vein.

ASHVC, the abdominal segment of the suprahepatic vena cava; PV, the portal vein between the right lobe branch and the gastroduodenal vein; TSHVC, the thoracic segment of the suprahepatic vena cava.

### Real-time Elastography

**Figure 2:**
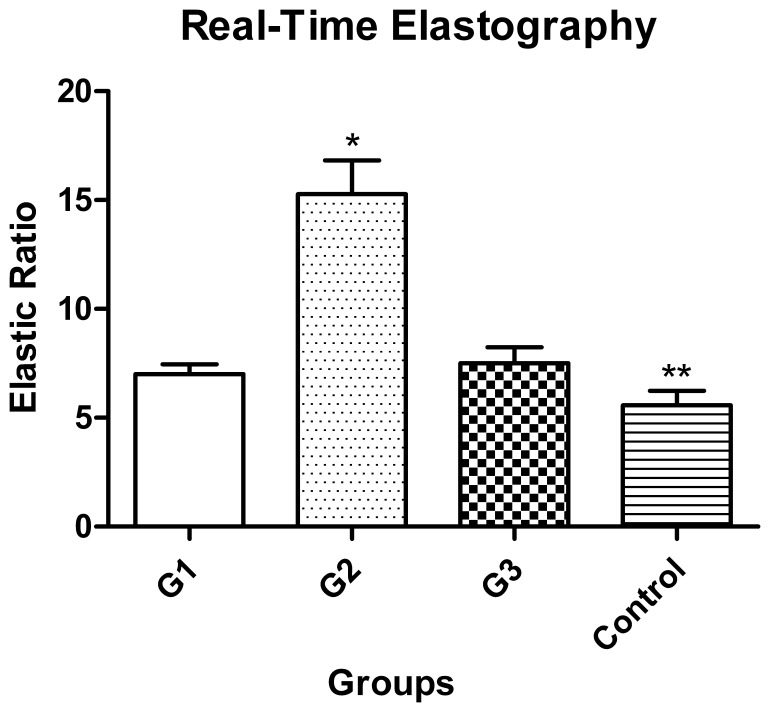
The elastic ratios of the transplantation groups and the control group. The elastic ratio in group 2 was the greatest, followed by group 1 and group 3. The elastic ratio in the control group was the lowest. There was no difference between group 1 and group 3. *Group 2 versus group 1, group 3, group 4 (P<0.01). **Control group versus group1 or group 3 (P<0.01).

In the present study, the mean elastic ratio was 5.57±0.68 in the normal rats. At the end of the one-month observation, the elastic ratios of the three transplantation groups were all higher than the control group (P<0.01), while the elastic ratio of group 2 was markedly higher than that of the other groups (P<0.01). The sharp contrast between these groups was shown in Figure 2.

### Hemodynamic Changes

In the control group, in which all the parameters were set as the normal value, the mean blood flow of the IHVC and PV were 26.08 ml/min and 12.83 ml/min, respectively. The pressure of the IHVC and PV were 2.31 mmHg and 9.82 mmHg, respectively. Twenty-four hours after the liver transplantation, the IHVC and PV blood flow were lower than the normal value, especially in group 2, which was the lowest. The pressure in all the IHVCs in the three groups was higher than normal. For the PV pressure, only in group 2 did it remain higher than the normal value.

**Figure 3:**
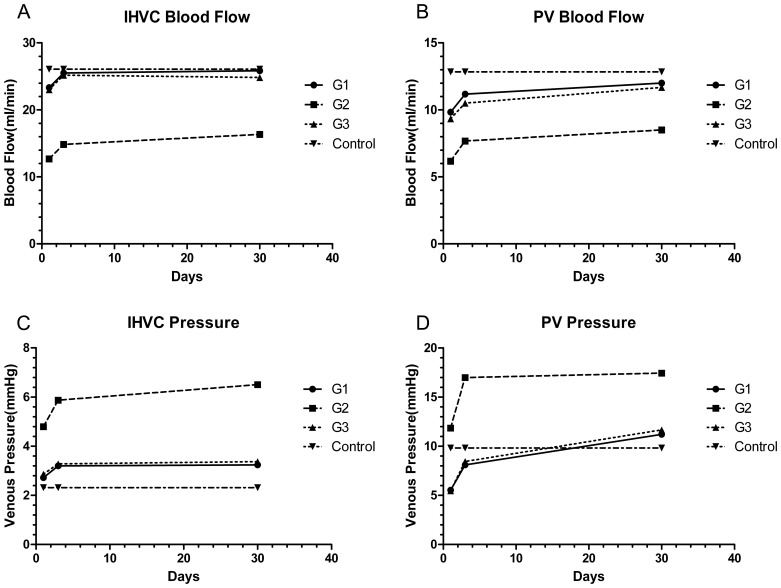
The IHVC and PV blood flow and pressure of the transplantation groups and the control group. The values in the control group were applied as normal value. The blood flow and pressure gradually increased in the transplantation groups. **A. and B.** All the IHVC and PV blood flow in the transplantation groups, especially in group 2, were below normal value during one month observation time (P<0.05). **C.** The IHVC pressure in the transplantation groups, especially in group 2, were higher than normal (P<0.05). **D.** The PV pressure in group 2 were higher than normal immediately following liver transplantation and then gradually increased (P<0.05), while that in group 1 and group 3 were lower than normal at 24 hr and 72 hr and were only slightly higher than normal at one month (P<0.05).

Three days and one month after the operation, the IHVC and PV blood flow were slightly lower than the normal value, except in group 2, in which it remained much lower than normal. During the same period, the IHVC and PV pressure gradually increased in the transplantation groups. One month after the operation, while the pressure in all the IHVCs and PVs was higher than normal, the pressure in group 2 was much higher than that in the other groups (Figure 3).

### Laser Speckle Perfusion Imaging

**Figure 4:**
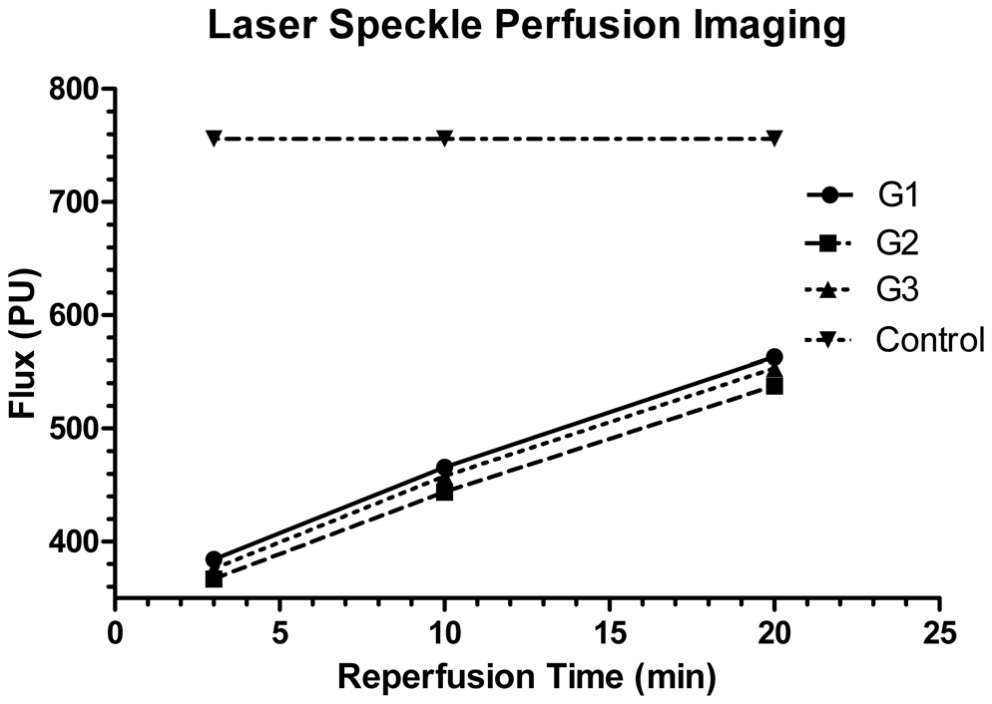
The laser speckle perfusion imaging of the grafted liver after reperfusion and the control. The flux value (PU) gradually increased in the three transplantation groups. The flux value in these groups was much lower than the control. There was no difference among the three transplantation groups (P>0.05).

The LSPI showed the surface microcirculation of the grafted liver with different reconstruction methods of the SHVC. According to our records, the flux value gradually increased after the reperfusion of the grafted liver (Figure 4), while there were no differences in the flux value among the three transplantation groups at 3 min, 10 min and 20 min after the reperfusion (P>0.05). The grafted liver was reperfused for 20 min, and the flux value remained lower than the normal value (P<0.05). This finding may reflect the reperfusion injury of the liver (Table 2).

**Table 2:** The flux value (PU) of the grafted liver after reperfusion and the controls.

Reperfusion Time (min)	Group 1#	Group 2#	Group 3#	Group 4*
3	384.4±9.7	367.0±34.5	375.9±10.2	755.7±75.6
10	465.7±10.8	443.8±28.3	457.8±15.8	N/A
20	563.0±21.1	537.5±50.7	551.2±35.3	N/A

# Group 1 versus group 2 versus group 3 (P>0.05);

* Versus group 1, 2, 3 (P<0.05).

### Venous Thrombosis

There were 11 (11/12), 9 (9/11) and 9 (9/11) survivors in group 1, group 2 and group 3, respectively, at one month after the operations. Among these survivors, there was no venous thrombosis (0/11) in group 1. The incidence of venous thrombosis was highest in group 2 (7/9). There were four cases of PV thromboses and three cases of IHVC thromboses. One animal had IHVC thrombosis and SHVC thrombosis. In group 3, the four cases of venous thromboses were in the SHVCs (4/9). The incidence of venous thrombosis in group 1 was lower than that in group 2 or group 3 (P<0.05). No significant difference of thrombosis-incidence was found between group 2 and group 3 (P>0.05).

### Time Expended on Each Step

**Figure 5:**
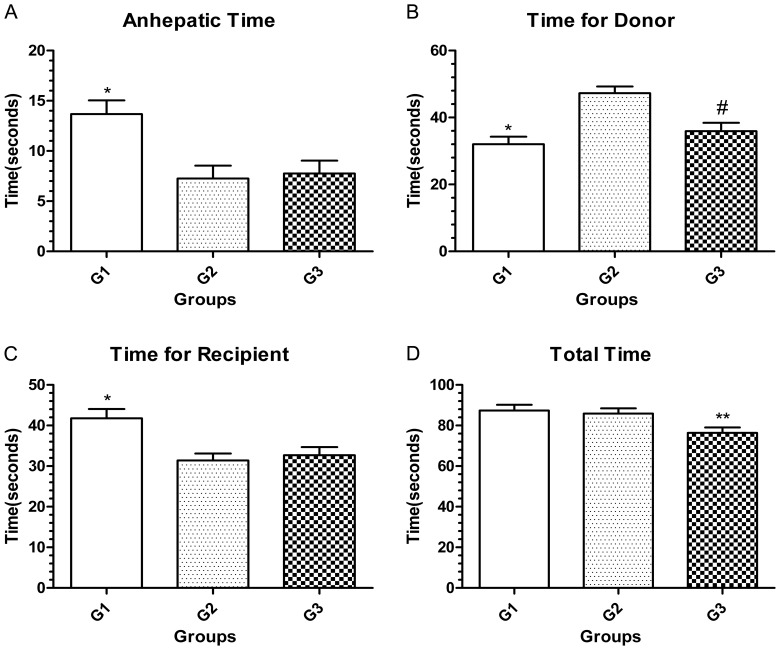
Details of the time-related metrics in each group. **A. and C.** Group 1 had the longest anhepatic time and recipient’s time. Group 2 and group 3 showed no significant difference in these two items. **B.** Group 1 expended the shortest time for the donor, followed by group 3. The time for the donor in Group 2 was the longest. **D.** Group 3 had the shortest total operation time. The total operation time between group 1 and group 2 were similar. * Versus group 2 or group 3 (P<0.05). # Versus group 2 (P<0.05). ** Versus group 1 or group 2 (P<0.05).

For the anhepatic time, Kamada’s technique in group 1 was longer than that in group 2 with the modified veno-lined stent technique or that in group 3 with Harihara’s cuff technique (P<0.05). No difference was found between the latter two groups (P>0.05). Kamada’s technique was the fastest for harvesting the graft liver, while the modified veno-lined stent technique was the slowest. For the recipient’s operation speed, Kamada’s technique was the slowest (P<0.05), and there was no difference between the other two techniques (P>0.05). The time for the total of the recipient’s and donor’s time was the total operation time. For this item, Harihara’s technique was approximately 10 min faster than the first two techniques (P<0.05) that had no significant difference between each other (P>0.05). The detailed time used on each step was shown in Figure 5.

### Survival Analysis

For the survival analysis in the animals, 34 totally successful arterialized ROLTs were performed. The success rates of liver transplantation were 100% (12/12), 91.7% (11/12) and 91.7% (11/12) in group 1, group 2 and group 3, respectively. There was no difference in the success rates between the three groups. During the first 24 hr after the surgery, two recipients died. One animal in group 2 died because of acute thrombosis of the SHVC. An animal in group 3 died because of slippage of the cuff in the SHVC.

There were no differences in the one week- and one month- survival rates among the three transplantation groups. In group 2, on the fifth day post-transplantation, one animal died because of thrombosis of the SHVC after the skew of the stent. In group 3, the slippage of the SHVC cuff led to the loss of one animal. One week after the transplantation, one animal died in group 1 because of obstruction of the biliary tract on the 13^th^ postoperative day, and a similar case in group 3 occurred on the 15^th^ day. In group 2, one animal died because of thrombosis of the SHVC on the 16^th^ day.

### Histological Analysis of the Survivors

Histological assessment was performed one month after the transplantation. The weight of the liver and spleen in group 1and group 3 were similar to that of the control group (P>0.05), while in group 2 it was much heavier (P<0.05). The majority of the livers in group 2 swelled markedly and showed a white and red appearance. The spleens were cruentous and much larger than normal (Table 3).

**Table 3:** Liver and spleen weight of the survival recipients and the controls.

	Liver weight (g)	Spleen weight (g)
Group 1#	12.1±1.0	1.0±0.06
Group 2*	15.7±1.1	1.4±0.09
Group 3#	12.4±0.8	1.0±0.07
Group 4#	12.0±0.9	1.0±0.1

# Group 1 versus group 3 versus group 4 (P>0.05).

* Versus group 1, 3, 4 (P<0.05).

**Figure 6:**
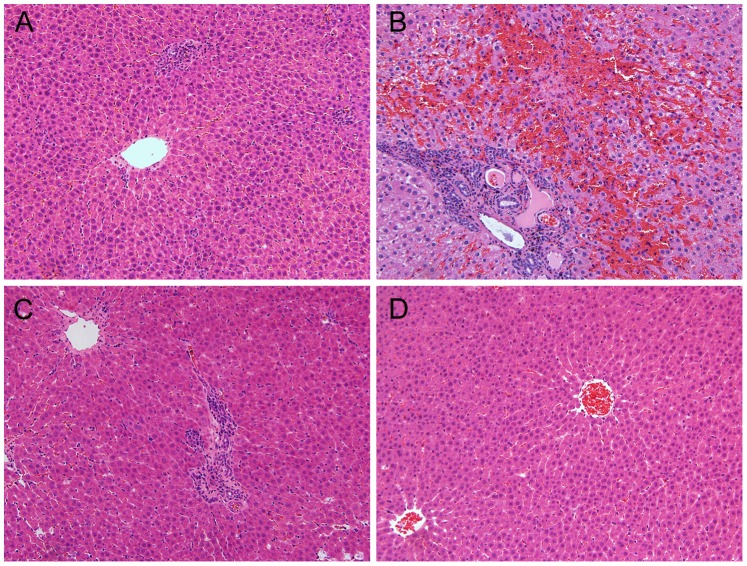
The typical pathological changes of the liver at one month in each group. **A. and C.** In group 1 and group 3, the hepatocytes were nearly normal. Mild cellular infiltration and ductular proliferation were seen. Minimal sinusoidal congestion and septal fibrosis were found. **B.** In group 2, extensive ductular proliferation presented in the area of the portal tracts. The hepatic sinuses dilated, and severe passive congestion was found within them. Necrosis of hepatocytes occurred to some extent in this area. **D.** In the control group, the hepatocytes were normal and the liver lobular architecture was intact.

The semi-quantitative analyses of the survivors’ livers were performed using the Mann-Whitney test. For cellular infiltration, sinusoidal congestion and ductular proliferation, the scores in group 2 with the modified veno-lined stent technique were much higher than those in the other two groups, while no significant difference was found for the scores of septal fibrosis among the three groups (Table 4). The typical histological changes of the survivors’ liver are shown in Figure 6.

**Table 4.The:** histological semi-quantitative score of the survivors’ livers.

	CI*	SC*	DP*	SF#
Group 1	1.45±0.69	0.73±0.46	1.36±0.81	1.91±0.83
Group 2	2.67±0.5	2.78±0.4	3.89±1.1	2.11±0.78
Group 3	1.44±0.88	1±0.7	1.56±0.89	2±0.87

* Group 2 versus group 1, 3 (P<0.05).

# Group 1 versus group 2 versus group 3 (P>0.05).

CI, cellular infiltration; DP, ductular proliferation; SC, sinusoidal congestion; SF, septal fibrosis.

## Discussion

There are at least three types of methods for reconstruction of SHVC, including the microsuture technique, cuff technique and veno-lined stent technique [1], [2], [3], [4], [5], [6]. There are few studies on the hemodynamics of rats after liver transplantation [7], [8], [9], [21], [22] and none that include the effects of SHVC reconstruction. In this experiment, we studied the anatomic basis of the transplantation-related veins and compared different SHVC reconstruction methods and their effects on the hemodynamics of the recipients. We found that the key point to performing a successful and stable ROLT was to ensure the amount and fluency outflow of the hepatic veins. Although the modified veno-lined stent technique using the TSHVC of the donor had a relative short anhepatic time and a similar one month survival rate, it led to markedly increased pressure and decreased blood flow of PV and IHVC and venous thromboses occurred more often. The techniques of Kamada and Harihara’s led to a minimal increase in portal pressure and did not significantly disturb the IHVC and PV dynamics and caused only a few venous thromboses, most likely because of outflow tracts were sufficiently wide.

### The Anatomic Basis for the Reconstruction of SHVC

For the ROLT, there was a consensus that the anastomosis of the IHVC and the PV were achieved by the cuff technique [23], [24], [25], [26], [27]. Although there are three different of methods, the reconstruction of the SHVC remains the most difficult part of ROLT.

From the anatomical study, we know that the average length of the ASHVC is 3.57 mm with a relative longer anterior wall and a shorter posterior wall. There are four phrenic veins that enter the ASHVC. The location of the left phrenic vein is variable, while the other three veins always join the ASHVC in the diaphragm. The left phrenic vein should be ligated in the donor and recipient. The upper end of the ASHVC is the phrenic ring, which crosses the vessel at an inclined plane. The lower end of the ASHVC is the junction of the hepatic veins of the median lobe and the left lateral lobe. The orifice of the upper end is an ellipse, while that of the lower end is an irregular circle.

When we use the microsuture method for reconstruction of the SHVC, to ensure that the anastomos is easy and safe, we must trim the donor’s and recipient’s SHVC into plain edges because the venous wall is so thin that every stitch should be made carefully and modestly. If the cuff technique is applied, appropriate tubes are very important for the TSHVC or the ASHVC. The diameter and length of the tubes must be correct for the ASHVC or the TSHVC [1], [3], [4].

When we use the modified veno-lined stent technique, the IHVC’s segment between the left renal vein and the left iliolumbar vein is used to cover the three-groove-stent. The branches of this vessel must be carefully ligated and cut off. This vessel is slightly wider than the TSHVC (3.13±0.18 mm vs. 2.82±0.12 mm). Differing from Tan’s method [5], the stent will be inserted into the TSHVC, and a 6 F stent will be sufficiently wide.

Tan’s method using the ASHVC was performed during the preliminary experiment. The results were dismal. Of the 12 recipients, the only two animals that had a successful liver transplantation died in one week. Most of the failures were caused by thromboses in the ASHVC or the hepatic veins. We hypothesized that the major reason was as follows. The donor’s IHVC for covering the polyethylene stent was much narrower than the donor’s ASHVC (3.13 mm vs. 6.76 mm) into which the veno-lined stent was inserted. When the ASHVC was ligated onto the veno-lined stent, a purse string effect appeared and there were many wrinkles in the ASHVC, inducing fatal venous thromboses.

### Hemodynamic Changes after ROLT

In rats after orthotopic liver transplantation, portal hypertension will not occur if all of the ASHVC, IHVC and PV are reconstructed by the microsuture method [7]. The total microsuture method for the ROLT is very difficult to master because it usually demands at least 12 months of carefully planned training to acquire the microsurgical technique [23]. Hence, the total microsuture method has never been widely used.

Kamada’s two-cuff technique and Harihara’s three-cuff technique were relatively simple to learn. Imamura and Kuznetsova found that these methods would cause a slight increase in portal pressure, which is a symptom of portal hypertension [7], [8].

The cuffs of the PV used in these two experiments (inner diameter, 1.42 mm or 1.67 mm) were apparently not wide enough for rats that weighed from 280 g to 400 g. In the present study, the inner diameter of the cuff for PV was 2.2 mm (for rats weighing from 320 g to 350 g). In our experience to gain enough perfusion of the graft, the inner diameter of the cuff must be at least 1.8 mm (for rats weighing approximately 200 g).

In Kuznetsova’s study, the PV blood flow was calculated but not directly measured, which would lead to bias to some extent. To exclude this potential error, we made the cuffs wide enough and directly measured the hemodynamic parameters by invasive methods.

One month after the operation, the modified veno-lined stent technique caused severe portal hypertension, while the techniques of Kamada and Harihara resulted in the portal pressure of the rats increasing slightly. Because all the PV cuffs were identical in the three groups, and there were no differences in the weight of the animals, we can conclude that the severe portal hypertension in group 2 was mainly caused by the veno-lined stent in the ASHVC.

From the anatomical study, we know that the IHVC for covering the stent was much narrower than the ASHVC. The outflow tract of the grafted liver was restricted after reperfusion, which not only increased the possibility of venous thrombosis but also induced the “relative obstruction” of the outflow tract. The sinus dilated, and sinusal congestion occurred in the grafted liver. The hepatocytes atrophied and necrosis appeared. Aseptic inflammation presented, and this effect led to cellular infiltration and ductular proliferation in the area of the portal tract. Simultaneously, portosystemic shunts appeared to compensate for the portal hypertension. Because the portal hypertension was aggravated, the portosystemic shunts could not ameliorate the portal hypertension effectively. The liver and spleen became swollen and presented the pathological appearance mentioned above (Table 3 and Figure 5B).

Although we made the outflow tract and the PV cuff as wide as possible in group 1 and group 3, a mild portal hypertension occurred. Similar to Kuznetsova and Imamura’s view [7], [8], we hypothesize that there would be hyperplasia of the grafted liver with the growth of the animals. However, the polyethylene-cuff restricted the enlargement of the PV. The stenosis would lead to a PV “ligature” effect. These human-induced changes would be “responsible” for the secondary portal hypertension of the recipients in group1 and group 3.

The cellular infiltration and ductular proliferation in group 1 and group 3 at one month were much less significant than that in Imamura’s study (Figure 5A and C). We hypothesize that the PV cuffs used in the present study were much more suitable than Imamura’s. The relatively wider PV cuffs provided a more effective blood supply to the grafted liver. There would be fewer portosystemic shunts and the portal hypertension would be gradually ameliorated. The portal hypertension in group 1 and group 3 was slight, and the pathological structure of the liver was the same as that of the control group (Figure 5D).

In this study, the real-time elastography had been used to evaluate the hepatic fibrosis and portal hypertension. The level of the portal hypertension was reflected by the liver stiffness (hepatic elastic ratio). From the study, we know that the hepatic elastic ratio in group 2 was the greatest. These results agree with the hemodynamic data and the pathological analysis that the portal hypertension in group 2 was the most severe. We found that even the mild portal hypertension in group 1 and group 3 could be distinguished from that in the control group by the real-time elastography method. Real-time elastography can precisely reflex the stiffness of the grafted liver.

There was no difference for the LSPI among the three liver transplantation groups. We consider that this result may be caused by the following reasons. The three groups used the same PV cuffs, which provided a similar PV blood supply. Because there were postoperative intraabdominal adhesions in the recipients, we only measured the LSPI after the reperfusion of the grafted liver. As there was reperfusion injury of all the grafted livers in the recipients, the LSPI in the three groups were lower than in the control group.

### Evaluation of the Modified Veno-Lined Stent Technique

In this study, we evaluated the veno-lined stent technique for the first time. There were a number of modifications of Tan’s veno-lined stent technique. First, the donor’s TSHVC was selected rather than the ASHVC because the latter was unsuitable for reconstructing of the SHVC due to the purse string effect mentioned above. Second, there was no similar process or extension on the end of the veno-lined stent, which looked like the structure of Miyata’s or Kamada’s cuff. Tan’s stent was very difficult to retain in an appropriate position in the operation of the donor or the recipient. Three circumferential grooves were carved on the stent, and these grooves were very helpful to retain the stent in a stable position.

Even with these modifications, the dissociating of the IHVC that was used to cover the stent was very difficult. The IHVC was thin and fragile. The IHVC should be dissected before the cold perfusion of the graft liver because of the filling with blood. In this condition, the dissection of the IHVC can be performed easily. The IHVC and the abdominal aorta must be separated meticulously because these vessels are too close to each other.

Although many details should be addressed, the modified veno-lined stent technique saved much anhepatic time compared to Kamada’s two-cuff technique, and it saved the recipient’s time. Because a number of additional steps were needed for preparing and fixing the stent in the donor operation, however, the graft-harvesting speed of the modified veno-lined stent technique was the slowest in the transplantation groups. When we calculate the total operation time, the modified veno-lined stent technique was longer than Harihara’s technique (shown in Figure 5).

As mentioned above, using the modified veno-lined technique restricted the outflow tract of the grafted liver, as in a model of the Budd-Chiari syndrome. The IHVC for covering the stent in our modified veno-lined stent technique was the same as in Tan’s method. The IHVC between the left renal vein and the left iliolumbar vein was applied these two methods. From our anatomical study we know that this segment of IHVC is slightly wider than the TSHVC, but it is much narrower than the ASHVC (3.13±0.18 mm vs. 6.76±0.67 mm). Even if Tan’s method could obtain a good long-term survival, it would severely restrict the outflow tract. Tan’s method did not obtain satisfactory result in our preliminary experiment.

In general, the ultimate aim of any modification of the classic method for ROLT was to maintain a healthy, stable and long-term survival of the animals. Although the animals in the modified veno-lined stent technique group gained the same one month-survival of those subjects of Kamada and Harihara’s technique, they had more venous complications and more severe portal hypertension. The modified veno-lined stent technique and Tan’s method for ROLT are not advised for the study of hemodynamics of animals after liver transplantation.

Whether we used the modified veno-lined stent technique or the three-cuff technique introduced by Miyata or Settaf [1], [2], the TSHVC was applied for reconstruction of the SHVC. In the former, the TSHVC was used for cover the lower end of the veno-lined stent. In the latter, the TSHVC was directly used for covering the cuff. From the anatomical study, we know that the TSHVC was much narrower than the ASHVC (2.82±0.12 vs. 6.76±0.67). The outflow obstruction of the grafted liver would appear because of the “new and constricted ASHVC” after liver transplantation. The venous complications and portal hypertension would occur gradually. We could infer that if the TSHVC is used for anastomosis of the SHVC either by the cuff or the modified veno-lined stent technique, there will be severe portal hypertension and venous complications. The modified veno-lined stent technique or the three-cuff technique introduced by Miyata or Settaf, which used the TSHVC, were not advised for hemodynamic studies.

## Conclusion

The hemodynamic changes after liver transplantation are affected by the PV cuff and are also determined by the SHVC cuff or stent. Sufficient venous outflow of the grafted liver is the key to ameliorate portal hypertension and decrease the occurrence of thromboembolic complications. The donor’s TSHVC is unsuitable for the reconstruction of SHVC. The modified veno-lined stent technique, Miyata’s or Settaf’s three-cuff techniques are not advised for hemodynamic studies, while Kamada’s two-cuff technique and Harihara’s three-cuff technique are very useful despite the presentation of mild portal hypertension.

## References

[1] Miyata M, Fischer JH, Fuhs M, Isselhard W, Kasai Y (1980) A simple method for orthotopic liver transplantation in the rat. Cuff technique for three vascular anastomoses. Transplantation 30: 335–338. 7006166 10.1097/00007890-198011000-00005

[2] Settaf A, Gugenheim J, Houssin D, Bismuth H (1986) Cuff technique for orthotopic liver transplantation in the rat. A simplified method for the suprahepatic vena cava anastomosis. Transplantation 42: 330–331. 3529537

[3] Tsuchimoto S, Kusumoto K, Nakajima Y, Kakita A, Uchino J, et al (1988) Orthotopic liver transplantation in the rat. A simplified technique using the cuff method for suprahepatic vena cava anastomosis. Transplantation 45: 1153–1155. 3289159

[4] Harihara Y, Sanjo K, Idezuki Y (1992) A modified cuff technique for suprahepatic vena cava anastomosis in rat liver transplantation. Transplantation 53: 707–709. 1549877

[5] Tan F, Chen Z, Zhao Y, Liang T, Li J, et al (2005) Novel technique for suprahepatic vena cava reconstruction in rat orthotopic liver transplantation. Microsurgery 25: 556–560. 16178005 10.1002/micr.20161

[6] Kamada N, Calne RY (1983) A surgical experience with five hundred thirty liver transplants in the rat. Surgery 93: 64–69. 6336859

[7] Kuznetsova LV, Zhao D, Wheatley AM (1995) Effect of orthotopic transplantation of liver on systemic and splanchnic hemodynamics in conscious rat. Am J Physiol 269: G153–159. 7631794 10.1152/ajpgi.1995.269.1.G153

[8] Imamura H, Rocheleau B, Cote J, Huet PM (1997) Long-term consequence of rat orthotopic liver transplantation with and without hepatic arterial reconstruction: a clinical, pathological, and hemodynamic study. Hepatology 26: 198–205. 9214470 10.1002/hep.510260126

[9] Wong J, Zhang Y, Lee SS (2001) Hemodynamic characterization of arterialized and nonarterialized liver transplants in the rat. Can J Gastroenterol 15: 435–440. 11493948 10.1155/2001/190508

[10] Kilkenny C, Browne WJ, Cuthill IC, Emerson M, Altman DG (2010) Improving Bioscience Research Reporting: The ARRIVE Guidelines for Reporting Animal Research. Plos Biology 8. 10.1371/journal.pbio.1000412 PMC2893951 20613859

[11] Higgins GM, Anderson RM (1931) Experimental pathology of the liver: restoration of the liver in the white rat following partial surgical removal. Arch Pathol 12: 186–202.

[12] Friedrich-Rust M, Ong MF, Herrmann E, Dries V, Samaras P, et al (2007) Real-time elastography for noninvasive assessment of liver fibrosis in chronic viral hepatitis. AJR Am J Roentgenol 188: 758–764. 17312065 10.2214/AJR.06.0322

[13] Friedrich-Rust M, Schwarz A, Ong M, Dries V, Schirmacher P, et al (2009) Real-time tissue elastography versus FibroScan for noninvasive assessment of liver fibrosis in chronic liver disease. Ultraschall Med 30: 478–484. 19813157 10.1055/s-0028-1109488

[14] Kanamoto M, Shimada M, Ikegami T, Uchiyama H, Imura S, et al (2009) Real time elastography for noninvasive diagnosis of liver fibrosis. J Hepatobiliary Pancreat Surg 16: 463–467. 19322509 10.1007/s00534-009-0075-9

[15] Koizumi Y, Hirooka M, Kisaka Y, Konishi I, Abe M, et al (2011) Liver fibrosis in patients with chronic hepatitis C: noninvasive diagnosis by means of real-time tissue elastography–establishment of the method for measurement. Radiology 258: 610–617. 21273523 10.1148/radiol.10100319

[16] Colombo S, Buonocore M, Del Poggio A, Jamoletti C, Elia S, et al (2012) Head-to-head comparison of transient elastography (TE), real-time tissue elastography (RTE), and acoustic radiation force impulse (ARFI) imaging in the diagnosis of liver fibrosis. J Gastroenterol 47: 461–469. 22223175 10.1007/s00535-011-0509-4

[17] Ochi H, Hirooka M, Koizumi Y, Miyake T, Tokumoto Y, et al (2012) Real-time tissue elastography for evaluation of hepatic fibrosis and portal hypertension in nonalcoholic fatty liver diseases. Hepatology 56: 1271–1278. 22488593 10.1002/hep.25756

[18] McGuire PG, Howdieshell TR (2010) The importance of engraftment in flap revascularization: confirmation by laser speckle perfusion imaging. Journal of Surgical Research 164: e201–212. 20863524 10.1016/j.jss.2010.07.059

[19] Blain B, Zhang F, Jones M, Richards L, Fischer K, et al (2001) Vascular grafts in the rat model: an anatomic study. Microsurgery 21: 80–83. 11372067 10.1002/micr.1014

[20] Zhao D, Zimmermann A, Wheatley AM (1993) Morphometry of the liver after liver transplantation in the rat: significance of an intact arterial supply. Hepatology 17: 310–317. 8428730

[21] Cao H, Wu Z, Zhang X, Zhang H, Chen Z, et al (2000) Changes in systemic and splanchnic hemodynamics after orthotopic liver transplantation in cirrhotic rats. Chin Med J (Engl) 113: 1108–1111. 11776147

[22] Cao H, Wu Z, Zhang X, Zhang H, Chen Z, et al (2003) The role of vasoactive substances in hyperhemodynamics after orthotopic liver transplantation in cirrhotic rats. Chin Med J (Engl) 116: 405–409. 12781047

[23] Holzen JP, Palmes D, Langer M, Spiegel HU (2005) Microsurgical training curriculum for learning kidney and liver transplantation in the rat. Microsurgery 25: 614–623. 16281279 10.1002/micr.20174

[24] Hori T, Nguyen JH, Zhao XD, Ogura Y, Hata T, et al (2010) Comprehensive and innovative techniques for liver transplantation in rats: A surgical guide. World Journal of Gastroenterology 16: 3120–3132. 20593497 10.3748/wjg.v16.i25.3120 PMC2896749

[25] Delriviere L, Gibbs P, Kobayashi E, Goto S, Kamada N, et al (1996) Detailed modified technique for safer harvesting and preparation of liver graft in the rat. Microsurgery 17: 690–696. 9588714 10.1002/(SICI)1098-2752(1996)17:12<690::AID-MICR6>3.0.CO;2-R

[26] Delriviere L, Gibbs P, Kobayashi E, Goto S, Kamada N, et al (1998) Technical details for safer venous and biliary anastomoses for liver transplantation in the rat. Microsurgery 18: 12–18. 9635788 10.1002/(sici)1098-2752(1998)18:1<12::aid-micr4>3.0.co;2-w

[27] Ishii E, Shimizu A, Takahashi M, Terasaki M, Kunugi S, et al (2013) Surgical technique of orthotopic liver transplantation in rats: the kamada technique and a new splint technique for hepatic artery reconstruction. J Nippon Med Sch 80: 4–15. 23470801 10.1272/jnms.80.4

